# Measuring the Market Power of China's Medical Product Exports

**DOI:** 10.3389/fpubh.2022.875104

**Published:** 2022-03-31

**Authors:** Wanshan Wu, Hao Zhang, Leping Huang, Lijun Wang

**Affiliations:** ^1^School of Economics, Zhejiang University of Technology, Hangzhou, China; ^2^School of Foreign Languages, Shenzhen University, Shenzhen, China

**Keywords:** market power, medical products, China, COVID-19, pandemic

## Abstract

During the COVID-19 pandemic, medical products have been crucial to the global fight against the disease. As a major manufacturing country, China occupies an important position in the medical products field. However, China's terms of trade are not commensurate with its status as a major exporter of medical products. Therefore, studying China's market power in medical product exports has important practical significance for determining China's value chain position in the global market and then proposing policies and measures to enhance China's market power. The findings of this paper, utilizing HS 6-digit data from 1992 to 2020, illustrate that China's market power is only in limited medical product export markets. Accordingly, we propose countermeasures to enhance the market power of China's medical product exports.

## Introduction

Since the COVID-19 pandemic began, China's outbreak has been effectively controlled. From January 1, 2022, to January 31, 2022, China experienced only dozens of new cases per day as well as the gradual recovery of the medical product supply chain in China, which is crucial to overcoming the pandemic because China is the main provider of medical products around the world. According to WTO data, China became the largest exporter of COVID-19-critical medical products in 2020; the total export value was US$105 billion, which was approximately 2.8 times as much as 2019. China's share of medical product exports is as high as 28.7% in the first half of 2020, which is 2.4 times that of the second largest exporter, the United States. China currently accounts for approximately 20% of the global medical product market. However, medical product companies are facing increasing competition and price pressures amid the rapid growth of new manufacturers. Especially in recent years, the Chinese government's reforms to reduce medical costs have made hospitals more price-sensitive. The 90% drop in stent prices following China's introduction of a volume-based procurement system for high-value medical consumables is a case in point. Does this price and cost pressure indicate that China relies more on quantity than quality to compete in export markets?

China's share of the medical product global market has steadily increased, but the terms of trade for many exports have not improved commensurately. Furthermore, export prices for some products have fallen sharply due to an increase in export volumes. Consider hand sanitizer as an example: China's export volume in 2020 was 40.2 times that of 2016, but the price continues to fall, and the terms of trade are deteriorating. The lack of pricing power in the medical product market suggests that the added value of China's exports has not increased, and its status quo as a large trading country rather than a strong country must improve. Therefore, we ask the following: does China have pricing power in the medical product export market? To this end, we employed products in the HS 6-digit 902000 (Breathing appliances and gas masks) to investigate China's market power in medical product exports and to accordingly propose countermeasures to enhance China's export market power.

## Literature Review

Several studies have examined the existence and theoretical explanations of pricing-to-market behavior. Fitzgerald and Haller (1) utilized plant-level microdata to demonstrate that producers selling in domestic and export markets participate in specific types of pricing-to-market behavior. The findings suggest that when prices are sticky, the optimal degree of market pricing conditional on price adjustments depends on other factors, including the choice of invoice currency, the nature of price stickiness, the expected frequency of price adjustments, and demand and cost shocks. Bergin and Feenstra (2) utilize local currency pricing and market pricing to explain why real exchange rates continue to deviate from purchasing power parity. They believed that the translog preference function produces greater persistence than the standard CES (constant elasticity of substitution) norm and better explains manufacturers' behavior in choosing different prices according to market differences.

Other literature analyzes the causes and manifestations of market power. Dhanora et al. (3) argue that innovative activities provide opportunities for firms to create and maintain industry monopoly power. Innovative firms typically earn market power by creating differentiated products through product innovation or by increasing productivity through process innovation. We demonstrate an inverted U-shaped relationship between technological innovation and market power based on empirical findings from Indian pharmaceutical companies. Auer et al. (4) note that product quality and target market income level are important dimensions that affect a firm's market-based pricing power. In wealthier markets, more consumers are willing to pay for higher-quality products. High-quality companies face low elasticity and therefore charge higher prices for their products. Furthermore, high-quality firms' market power varies widely, and their markups are more responsive to exchange rate fluctuations than those of low-quality firms. The results demonstrate that the relative price of high-quality goods compared to inferior goods is an increasing function of income in the destination market. Therefore, low-quality goods are relatively more expensive in poor markets, while high-quality goods are relatively more expensive in wealthy markets.

Other scholars have explored the impact of market power and how it is ideally addressed. Wang et al. (5) utilized unique matched data from SME (small and medium enterprises)-bank relationships in 19 European countries to examine the impact of bank market power on SME financing. Research suggests that bank market power reduces SMEs' access to bank financing. Consequently, to reduce the adverse effects of market power while promoting competition among banks, policymakers must also support SMEs' access to financing by reducing information barriers and building tailored relationships. Mertens (6) utilizes 20 years of German manufacturing microdata to explore the reasons for the global decline in labor's share of economic output, arguing that the firms' market power in labor and products explains half of the decline in labor shares. Elsayed et al. (7–9) discussed economic uncertainty, COVID-19, and labor market regulations. Chen et al. (10–13) also examined the effects of conflict, social mobility and stringency measures on COVID-19 and the economy.

## Feature Facts and Statistical Descriptions

Breathing appliances and gas masks are essential products for controlling COVID-19; hence, we selected representative products in the HS 6-digit 902000 to examine China's market power in the medical product market. The data come from the United Nations' Comtrade database. Data such as nominal exchange rates and price indices come from the World Bank's World Development Indicators and the CEIC's global economic data, indicators, charts, and forecasts as well as other databases.

**Figure 1:**
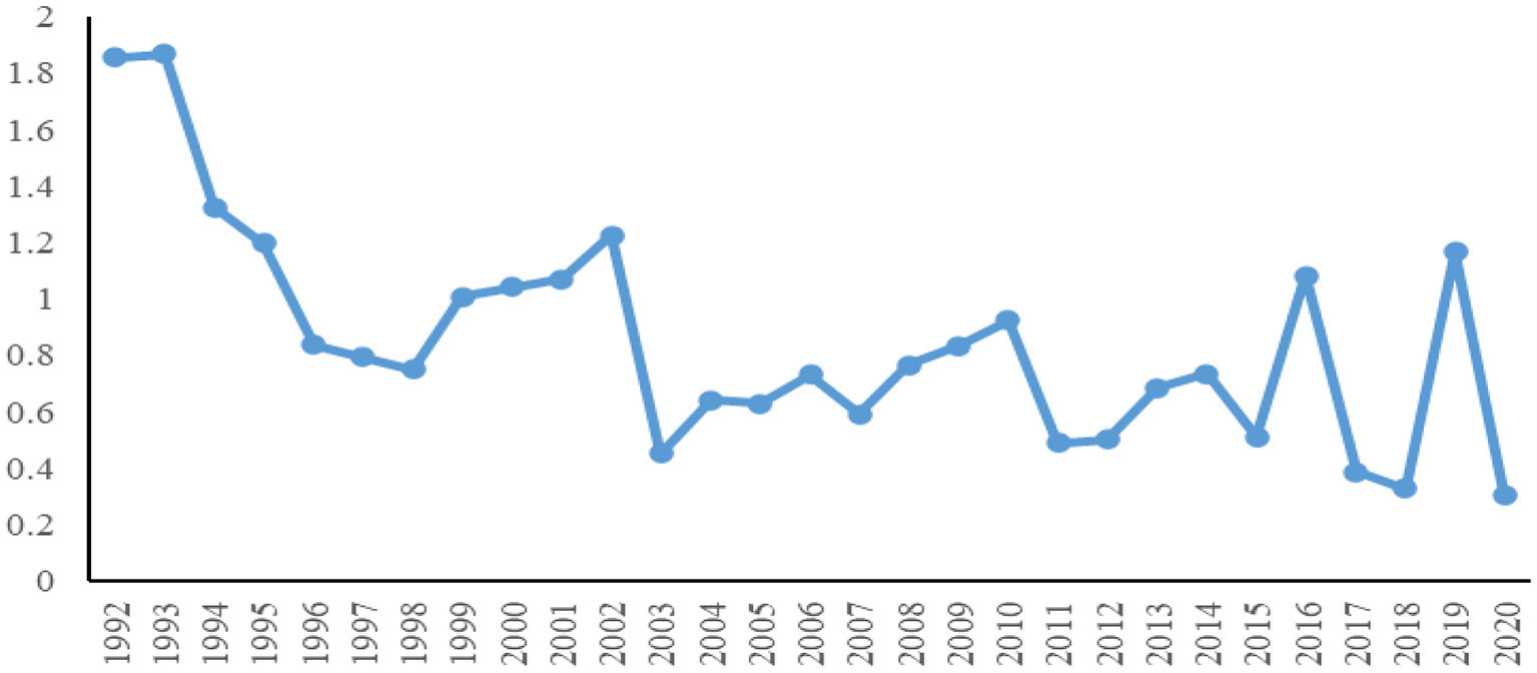
1992–2020 China's trend in terms of trade for breathing appliances and gas masks.

We employed the 902000 product code to examine the changing trend of its terms of trade to understand the characteristics and facts of China's pricing power in this product market. Based on the data from Comtrade, we calculated the terms of trade for breathing appliances and gas masks. The calculation results indicate that the overall terms of trade for this medical product feature a gradual deterioration trend (see Figure 1), which illustrates that China has not gained pricing power commensurate with its market share in this product market.

The statistical description of the product category reveals that the maximum export price for China's breathing appliances and gas masks was 27.4, obtained in Malaysia in 2013 (see Table 1). The minimum value was 0.14, obtained in the Philippines in 1998. The maximum value of the nominal exchange rate was 3482.8, obtained in Vietnam in 2015, and the minimum value was 0.066, achieved in the United Kingdom in 2007. The maximum real exchange rate was 3595.2, achieved in Vietnam in 2001, and the minimum was 0.04, achieved in the United Kingdom in 2004.

**Table 1:** Statistical description of the main variable.

	**Price**	**Nominal**	**Real**
Mean	4.213	122.849	143.324
Median	3.417	0.202	0.220665
Maximum	27.447	3,482.792	3,595.236
Minimum	0.144	0.066	0.044
Observations	602	602	602

## Empirical Research

We propose an extension model of China's market power in the medical product export market based on Knetter's (1989) model. In the market power model, the measurement of market power is based on the Lerner index, and the pricing-to-market parameter is utilized to reflect the size of the market power. Pricing-to-market means that when the currency of the importing country appreciates, the exporter does not lower the price but maintains or increases the price of the export commodity. This reflects exporters' market power.

If a country's manufacturer exports medical products to *n*independent foreign target markets and each market is denoted by *i*, then the exporter's profit function is as follows:


(1) $$\begin{array}{c} {\prod^{\text{​}}}_{t}=\sum^{\text{​}}p_{it}q_{it}(e_{it}p_{it},v_{it})-C_{t}(\sum^{\text{​}}q_{it}(e_{it}p_{it},v_{it}),\eta_{t})\\ \;i=1,...,n \end{array}$$

In Equation 1, *p*_*it*_represents the price of export to country *i* at time *t*in the currency of the exporting country, *q*_*it*_is the demand of the importing country at time *t*, *e*is the exchange rate (the importing country/ the exporting country), and is the cost function under profit-maximizing conditions, we can get:


(2) $$\begin{array}{r} P_{it}=MC_{t}\left(\frac{E_{it}}{E_{it}-1}\right) \end{array}$$

In Equation 2, *E*_*it*_represents the price elasticity of demand in importing country *I*, which is a function of exchange rate *e*; represents the marginal cost of medical products. Equation 2 illustrates that the price expressed in the currency of the exporting country is a markup on marginal cost. The size of the markup depends on the price elasticity of demand in each target market. Employing the natural logarithm of Equation 2 provides an empirical model for the pricing-to-market parameter:


(3) $$\begin{array}{r} \ln\;P_{it}=\theta_{t}+\lambda_{i}+\beta_{i}\ln\;e_{it}+u_{it} \end{array}$$

Here, θ_*t*_, λ_*i*_, β_*i*_, and represent time effect, country effect, the pricing-to-market parameter, and the random disturbance term, respectively. Among them, time effect is θ_*t*_, which is utilized to measure the change in marginal cost. Target market effect is λ_*i*_, which represents the size of the markup; in other words, it is the degree to which the export price deviates from the marginal cost in different target markets. The pricing-to-market parameter β_*i*_ indicates the extent to which exporters adjust their export prices in local currency when exchange rates fluctuate. It reflects exporters' ability to differentiate pricing according to market differences and is also the level of market power.

Assume that the market of the importing country is perfectly competitive, that is, the price is equal to the marginal cost, and the export price is equal in different target markets. Time effect θ_*t*_in Equation 3 represents the constant price in each period. Additionally, λ_*i*_ and β_*i*_ are equal to 0 in a perfectly competitive importing country's market.

We assume that the importing country's market is a monopoly market with price discrimination, but the price elasticity of demand in the importing country remains unchanged. The price markup in Equation 2 reveals that constant elasticity means that the export price in a specific target market has a fixed markup λ_*i*_ relative to the marginal cost in Equation 3, but the markup may vary depending on the target market. The key implication of a price discrimination model with constant elasticity of demand is that residual changes in export prices are independent of the exchange rate of a particular target market. Therefore, the invariant elasticity assumption implies that β_*i*_ is 0.

We assume that the importing country's market is a monopoly market with price discrimination but that the importing country's price elasticity of demand is constantly changing. Equation 2 demonstrates that the constant change of elasticity means that markup λ_*i*_ is constantly changing in each target market in Equation 3. Therefore, when exchange rate *e* changes, there is a price difference between export price *p*_*it*_ denominated in the exporter's local currency and price *e*_*it*_*p*_*it*_ paid by the importing country in the importing country's currency. Exporters must adjust the markup for their interests: export price *p*_*it*_ should change with exchange rate *e* so the pricing-to-market parameter β_*i*_ is not 0.

**Figure 2:**
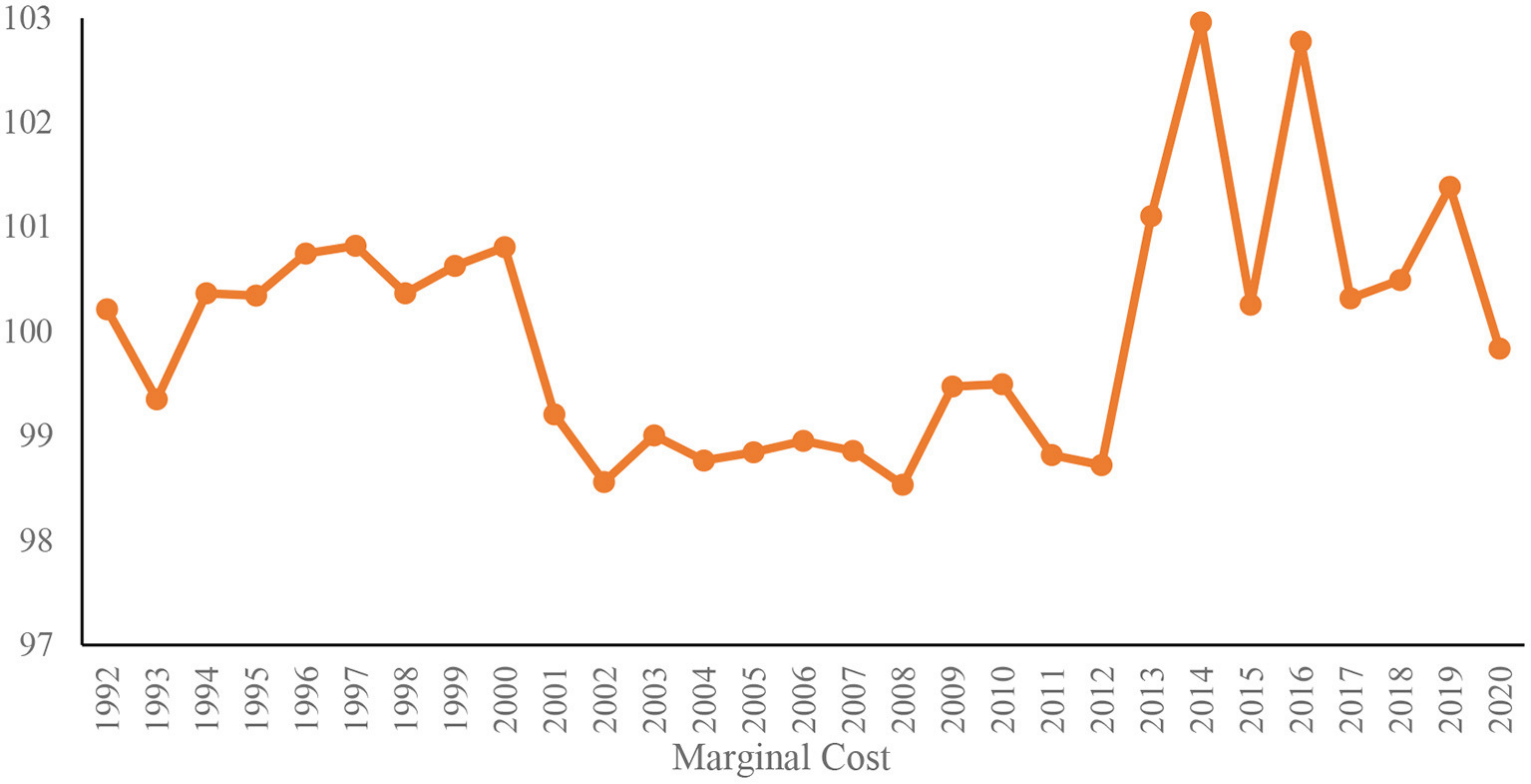
Trend of the marginal cost of medical products in China (HS: 902000).

The target market effect parameter λ_*i*_and the pricing-to-market parameter β_*i*_ can be employed to determine the market power level of China's medical product exports. Utilizing data from 21 target markets, we examined the market structure and power of China's medical product exports. Each export market is estimated utilizing nominal and real exchange rates. The empirical results are presented in Table 2 and Figure 2.

**Table 2:** Chinese medical products' (HS: 902000) market power status in different countries.

**Targeted market**	**Nominal exchange rate**		**Real exchange rate**	
	**λ_*i*_**	**β_*i*_**	**λ_*i*_**	**β_*i*_**
Australia	−3.06	22.94	9.49	16.14^*^
Canada	0	21.12	−5.9	21.60^**^
France	−1.12	−4.61	−1.48	−4.89
Germany	−6.13	−2.63	−2.6	−6.46
Greece	9.19	−36.02	−1.38	−12.01
Israel	−6.83	−20.40^***^	−1.34	−8.56^**^
Italy	−1.36	1.93	−9.34	−2.12
Japan	3.52	−0.03^**^	7.04	0.02^***^
Korea	−4.13	0.023	−2.3	0.01^**^
Malaysia	3.19	18.61	−1.23	8.65
Netherlands	−4.2	−38.17	−1.84	−21.53
New Zealand	1.15	33.50	1.16	16.79
Philippines	−1.86	0.02	−8.75	−0.06
Russia	5.18	0.36^**^	−2.61	0.23^**^
Saudi Arabia	−5.97	6.36	−1.65	1.17
Singapore	−4.78	8.13	−2.59	32.32
Spain	−3.68	25.39	−5.51	5.99
UAE	1.42	14.23	2.63	10.19
UK	−2.37	−29.23	−9.49	−5.23
US	−1.01	22.25^*^	−7.43	6.22
Vietnam	−3.22	0.003^***^	4.25	−0.0006

*, **, *** *Indicates statistical significance at the 10%, 5% and 1% level*.

The research results in Table 2 indicate that in almost all Chinese medical product export markets, the target market effects measured by nominal and real exchange rates are significantly different from 0. This suggests that the null hypothesis of China's perfect competition in the medical product export market does not hold true. Additionally, it means that the law of one price caused by commodity arbitrage does not hold true, and China is a non-perfectly competitive market in the medical product export market. This is closely related to the fact that the medical product export market is primarily dominated by China, the United States, and Germany.

Calculations utilizing real exchange rates demonstrate that the null hypothesis of constant elasticity of demand is rejected in medical product export markets such as Israel, Japan, Russia, the United States, and Vietnam. Consequently, in the majority of China's medical product exports, the real exchange rate reflects inflation fluctuations, and changes in exchange rates do not lead to corresponding changes in export prices. The calculation results utilizing the nominal exchange rate indicate that the null hypothesis of rejecting the constant elasticity of demand is only valid in Australia, Canada, Israel, Japan, South Korea, and Russia. Therefore, when the exchange rate fluctuates among the 21 export markets, China has the ability to adjust prices in only a few export markets; in other words, its market power is limited. This is significantly disproportionate to China's top position in the world's medical product export market and suggests that utilizing market share to measure China's market power in the medical product market is misleading. The potential market power indicated by China's market share in the medical product market does not match its actual market power, which demonstrates that China lacks pricing power in the medical product export market. China remains a sizable country rather than a power in the medical product export market. Measurements utilizing real exchange rates illustrate that among countries that reject the null hypothesis of constant elasticity of demand, only the Israeli market has a negative price-to-market parameter, which suggests that Chinese medical product exporters are attempting to stabilize product prices in this market.

Figure 2 depicts the coefficient of the time effect in the regression equation estimated by the real exchange rate from 1992 to 2020, that is, the trend of the marginal cost of China's medical product exports. Empirical research results indicate that the marginal cost index of China's medical product exports has risen sharply since 2007, which is related to the promulgation of China's labor contract law. The increase in labor costs directly augmented the marginal cost of medical products. Accordingly, China's share of the worldwide medical product export market has continued to rise, although the terms of trade have been deteriorating. This is related to China's reliance on quantity rather than quality competition in the medical product export market. Chinese medical product exporters are typically small-scale, low-value-added companies with limited market power. Due to the large number of Chinese exporters, these companies are forced to reduce prices to gain market share, which has kept China at the bottom of the smile curve in the medical product export market.

## Conclusions and Policy Recommendations

We employed the HS 6-digit 902000 to investigate the market structure of China's medical product export market and China's market power. The empirical research results reveal that China's medical product export market is a non-perfectly competitive market. The law of one price caused by commodity arbitrage does not hold true in China's medical product export market. Among the 21 markets to which Chinese medical products are exported, only a small number, including Israel, Japan, Russia, the United States, and Vietnam, reject the null hypothesis of constant demand elasticity, which illustrates that China has limited market power in the international medical product export market and thus lacks pricing power. This is disproportionate to China's position as a global leader in medical product exports. In the Chinese medical product export market, the Israeli market, which rejects the null hypothesis of constant demand elasticity, has a negative pricing-to-market parameter, indicating that Chinese medical product exporters are attempting to stabilize market prices in the country. To change the status quo—China has market share advantages but no market power in some medical product markets—the nation must strengthen R&D as well as production of high-end medical equipment, such as ECMO and medical artificial intelligence microchips, to effectively enhance the added value and market power of exported medical products and eliminate problems at the lower end of the value chain.

## Data Availability Statement

Publicly available datasets were analyzed in this study. This data can be found at: https://comtrade.un.org/data/.

## Author Contributions

WW: writing—original draft. HZ: review and editing. LH: proofreading. LW: investigation and software. All authors contributed to the article and approved the submitted version.

## Conflict of Interest

The authors declare that the research was conducted in the absence of any commercial or financial relationships that could be construed as a potential conflict of interest.

## Publisher's Note

All claims expressed in this article are solely those of the authors and do not necessarily represent those of their affiliated organizations, or those of the publisher, the editors and the reviewers. Any product that may be evaluated in this article, or claim that may be made by its manufacturer, is not guaranteed or endorsed by the publisher.
